# American Medical Monthly

**Published:** 1863-04

**Authors:** 


American Medical Monthly.—We regret to see it announced in the December number of this journal that its publication will be suspended. "The sole cause of this suspension is the continued absence of the editor, which prevents him from giving any attention to his editorial duties, or—as he holds the title of the journal—from supervising its business arrangements."

"With the cessation of hostilities, with retiring war, with returning peace, the editor hopes to resume his labors, and he confidently trusts that the pleasant relations which for many years have existed between himself and the editorial corps of his profession, and the subscribers to the *Monthly*, may be speedily and happily renewed."

